# Injectable light-assisted thermo-responsive methylcellulose-sodium humate hydrogel proposed for photothermal ablation and localized delivery of cisplatin

**DOI:** 10.3389/fbioe.2022.967438

**Published:** 2022-08-08

**Authors:** Farnaz Ghorbani, Behafarid Ghalandari, Zichen Liu, Dejian Li, Baoqing Yu

**Affiliations:** ^1^ Department of Orthopedics, Shanghai Pudong New Area People’s Hospital, Shanghai, China; ^2^ Department of Orthopedics, Shanghai Pudong Hospital, Fudan University Pudong Medical Center, Shanghai, China; ^3^ State Key Laboratory of Oncogenes and Related Genes, Institute for Personalized Medicine, School of Biomedical Engineering, Shanghai Jiao Tong University, Shanghai, China; ^4^ School of Materials Science and Engineering, University of Shanghai for Science and Technology, Shanghai, China

**Keywords:** photothermal therapy, chemotherapy, local delivery, protein interaction, light-assisted thermo-responsive hydrogel

## Abstract

This study aimed to develop injectable light-assisted thermo-responsive methylcellulose hydrogels filled with sodium humate, which were proposed for photothermal ablation and localized cisplatin delivery. Sodium humate converts light energy from laser beams into thermal energy, which causes methylcellulose to gel, thereby controlling the release of chemotherapy agents. Meanwhile, light emission causes to the photothermal ablation of tumor cells. For determining the optimal production conditions, different concentrations of sodium humate and light emission times were investigated. Results show that hydrogel uniformity is highly dependent on variables. An increase in sodium humate concentration and emission time resulted in a slight reduction in swelling ratio and an increase in durability. According to the simulation conditions, the cisplatin release profile was consistent with a non-Fickian mechanism with a predominant erosion contribution. In conjugation with increasing light emission time and sodium humate content, the storage modulus and viscosity increased, demonstrating hydrogel’s sol-gel transition and long-lasting durability. The intrinsic fluorescence spectroscopy study revealed that the hydrogel-model protein complex empowered hydrogel bio-performance. Laser emission and cisplatin release synergistically reduced the number of viable osteosarcoma cell lines, suggesting the possibility of tumor ablation. This study describes the potential of simultaneous photothermal therapy and chemotherapy in osteosarcoma treatment, laying the groundwork for future preclinical and clinical trials.

## 1 Introduction

Urgent treatment of cancer is a significant global concern. Conventional treatments such as systemic chemotherapy and radiotherapy have shown undesirable side effects and low therapeutic efficacy (Tang et al., 2020; Xu et al., 2022). Localized drug delivery has attracted various scientists due to its lower toxicity and controllable release rate (Qin et al., 2021).

Polymeric matrices can be used for localized drug delivery. Herein, injectable cross-linked hydrogel carriers with the potential of mimicking the target tissue environments, biocompatibility, and biodegradability have been employed for *in-situ* delivery of pharmaceuticals with the highest level of bioavailability (Zhu et al., 2021; Kim et al., 2022). In this regard, Ma et al. (2015) introduced a new approach to osteosarcoma treatment by localized co-delivery of doxorubicin, cisplatin, and methotrexate which were encapsulated in thermo-sensitive poly lactic-co-glycolic acid/polyethylene glycol hydrogels. Thermosensitive hydrogels allows for easy mixing of pharmaceutical agents and liquid-like polymers at ambient temperature, whereas drug-loaded free-standing hydrogels can be formed upon injection of the drug at body temperature. Drug-loaded hydrogels exhibited cytotoxicity against osteosarcoma Saos-2 and MG-63 cells, *in-vivo* expression of tumor apoptosis-related genes, and the most significant tumor suppression.

In addition to the many polymers that have been adapted as injectable hydrogels and therapeutic carriers, methylcellulose, a methylated cellulose, has been widely applied in pharmaceutics. Methylcellulose display thermo-responsive behavior. Thermo-responsive methylcellulose-based hydrogels have shown a high level of viscosity and suitable shear-thinning property at ambient temperature. Accordingly, *in-situ* gelation at elevated temperatures is preferred for medical applications. Since this phenomenon occurs with the dehydration of methylcellulose, hydrogels can be used for long-term delivery of pharmaceutical agents (Liu and Yao, 2015). To full-fill this aim, it is essential to increase the temperature of methylcellulose hydrogel more than low critical solution temperature (LCST) (∼50°C) in order to bind free polymer chains and make interconnected network in order to physical gelation (Bonetti et al., 2021; Coughlin et al., 2021). However, a consideration should be made that heating is not the only factor affecting the physical cross-linking of methylcellulose. A number of factors, including methylcellulose concentration, methylcellulose molecular weight, external stimuli, temperature ramp, anions, and blending can affect the gelation process and tune the LCST response (Bonetti et al., 2021).

An important aspect of drug efficacy is the ability to control the release rate. Hydrogels capable of responding to stimuli factors such as pH, temperature, and light may be able to overcome burst-release problems (Abed et al., 2018; Ghorbani et al., 2019; Jo et al., 2022; Pedige et al., 2022). Herein, light-assisted thermo-responsive hydrogels are presented as sustainable drug carriers. Reversible phase transition of hydrogels under elevated temperature arising from light emission leads to controlled release behavior of constructs. Although various light-assisted thermo-responsive materials induce photothermal behavior such as Titanium dioxide nanocrystals (Yu et al., 2017), tellurite nanostructures (Yu et al., 2018), Chlorin e6 (Yu et al., 2021a, Yu et al., 2022), cytotoxic effects, light scattering, and non-degradability (Qin et al., 2016) have restricted their applications. In this regard, sodium humate has presented theranostic applications in photothermal therapy and local cancer treatment (Yu et al., 2021b). The great potential of this material lies in its ability to absorb and convert near-infrared light into thermal energy. Addition of sodium humate to the chemical composition of the gels can convert simple hydrogels into light-assisted thermo-responsive ones, which enable the gelation of methylcellulose by producing thermal energy and facilitate localized delivery of loaded agents as well as photothermal ablation of tumor cells.

Incorporating light-stimulating hydrogels with anti-tumor drugs can contribute to the synergistic effect of photothermal therapy and chemotherapy. Cisplatin has been shown to be highly effective at inhibiting various types of tumors, including osteosarcoma by cross-linking DNA in a number of different ways so as to interfere with mitosis and promote apoptosis (Luetke et al., 2014; Hou et al., 2022).

In this study, light-assisted thermo-responsive methylcellulose hydrogels containing sodium humate were prepared by raising the temperature as a function of laser illumination. Laser emission can also contribute to the overall ablation of tumor cells. An injectable hydrogel was used in conjunction with cisplatin to achieve simultaneous photothermal therapy and long-term chemotherapy for osteosarcoma ablation. In this respect, we investigated the influence of light absorbance concentration and laser exposure duration. To make hydrogels more durable, silane coupling agents were used to chemically modify the methylcellulose. Afterward, the prepared hydrogels were subsequently characterized physicochemically and rheologically. Likewise, protein interactions have been examined by fluorescence spectroscopy, as well as cytotoxicity through cell viability studies. Finally, the optimal compositions for further preclinical and clinical examinations were introduced in order to achieve successful photothermal treatment and chemotherapy.

## 2 Materials and methods

### 2.1 Materials

Methylcellulose (viscosity: 15 cP), sodium humate (M_w_ 226.14 g/mol), cisplatin (M_w_ 300.05 g/mol), phosphate-buffered saline (PBS, tablet, pH 7.4), human hemoglobulin (HB), and Albumin from human serum (HSA) were purchased from Sigma-Aldrich Co. (Missouri, United States). (3-Glycidyloxypropyl)trimethoxysilane (GPTMS, M_w_ 236.34 g/mol) and sodium hydroxide (M_w_ 40 g/mol) were purchased from Rhawn Co. (Shanghai, China). All chemicals were utilized directly without further purification. Aqueous solutions were prepared with deionized (DI) water.

### 2.2 Preparation of hydrogels

The methylcellulose-based hydrogels were prepared in PBS with a concentration of 10% (w/v). To accomplish this, half of the total volume of PBS was heated to 90°C and the remaining amount cooled to 0°C. Heated PBS solution was used to dissolve the methylcellulose and chilled solution to dissolve the sodium humate. In a subsequent step, sodium humate solution was gradually added to the heated methylcellulose solution. Then, pH of the solution was adjusted to 9, and GPTMS with a weight ratio of 1:1 (GPTMS: methylcellulose) was added to the solution and aged for 2 h. Finally, the prepared solution was kept at 4°C overnight. The prepared solution was irradiated with laser light (808 nm, 2 W/cm^2^) for different times (5 and 10 min) for complete gelation and further investigation. Table 1 shows the details about the prepared test samples.

**TABLE 1:** Details regarding the preparation of hydrogels.

Code	Methylcellulose concentration, % (w/v)	Sodium humate concentration (mg/ml)	GPTMS: Polymer concentration (wt%)	Laser emission time (minutes)
MS1	10	2.5	1:1	5
MS2	10	5	1:1	5
MS3	10	5	1:1	10

### 2.3 Characterization of hydrogels

The morphology of the hydrogels was observed using light microscopy (CKX53, Olympus Co., Tokyo, Japan) in wet condition, and dried hydrogels were evaluated with field-emission scanning electron microscopy (FE-SEM, Nova NanoSEM 450, FEI Co., Oregon, United States).

Chemical characterization of the hydrogels was performed by Fourier transforms infrared spectrophotometer (FTIR, Nicolet Is50, Thermo Fisher Scientific, Massachusetts, United States) between 400 and 4,000 cm^−1^ with a resolution of 4.0 cm^−1^ and eight scans.

The cross-linking degree of hydrogels was calculated using Eq. 1 (Barkoula et al., 2008), where G′ is storage modulus, T is temperature, and R is gas constant. G′ was obtained from the linear region of frequency sweep test at 37°C ± 0.5°C.

$$\mathrm{Degree of cross-linking}\left(\%\right)=G^{\prime }/\mathrm{3RT*100}$$ (1)

Hydrogels-water molecules interactions were evaluated by calculation of swelling ratio and water content (Supplementary Information). So, the dry weight of hydrogels (Wi) and wet weight (Ww) after incubation of samples in the PBS solution at 37°C ± 0.5°C was measured at pre-determined time points (6, 12, and 24 h). Finally, the swelling ratio was calculated according to Eq. 2 (Zhang et al., 2020):

Swelling ratio(%)=[(Ww-Wi)/Wi]*100 (2)

Dissolution of hydrogels was followed after incubation of cross-linked species in the PBS solution at 37°C ± 0.5°C. Accordingly, the initial dry weight (Wi) and secondary dry weight (Ww) at pre-determined time points (1, 3, 6, 9, 12, and 15 days) were measured, and the dissolution ratio was calculated according to Eq. 3 (Al-Sibani et al., 2015):

Dissolution ratio(%)=|[(Ww-Wi)/Wi]|*100 (3)

The drug-loaded samples were prepared by dissolving the cisplatin with a concentration of 1.5 mg/ml in methylcellulose-sodium humate solution before laser emission (as explained in Section 2.2). Then, 0.5 ml of laser-irradiated samples were incubated in 3 ml of the PBS solution at 37°C ± 0.5°C. The concentration of released cisplatin was measured over time (1, 3, 6, 9, 12, and 15 days) using a UV-Vis spectrophotometer (UV-VIS, Lambd25, Perkin-Elmer Co., United States) at a wavelength of 207 nm. The hydrogels without cisplatin were served as blank samples.

The rheological characterization of hydrogels was done by a parallel plate rheometer (MCR300, Anton paar, Germany). The strain sweep test was carried out at a constant frequency (*ω*) of 1 rad/s and strain range of 1 < *γ* < 100% at constant temperature (37°C ± 0.5°C). Frequency sweep test was done at an angular frequency of 1 < *ω* < 100 rad/s and 10% strain at temperature 25 ± 0.5°C and 37 ± 0.5°C. Temperature sweep test was performed at a constant frequency (*ω*) of 1 rad/s and a temperature range of 30°C–60°C at a heating rate of 1°C/min.

### 2.4 Interaction of hydrogel with model proteins

The hydrogel interaction with HSA and HB as model proteins was studied by fluorescence spectroscopy using a Cary Eclipse spectrofluorometer (Varian, Australia). According to the characterization and *in vitro* release study, the best hydrogel was selected for the interaction studies. The various concentration of hydrogel (0–70 µM) were injected into the protein solutions (6 µM) at 25 and 37°C. The excitation wavelength was adjusted at 290 nm. The emission spectra were recorded at 300–500 nm after 5 min of incubation time.

### 2.5 Cell viability

Behavior of MS2 hydrogels against osteosarcoma cell lines (Saos-2 and MG-63) was evaluated by MTT (3-{4,5-dimethylthiazol-2yl}-2,5-diphenyl-2H-tetrazolium bromide) assay. Briefly, 50 µl MS2 hydrogel was incubated with 5 × 10^4^ cells/well for 48 h in DMEM supplemented with 10% FBS, 100 g/ml penicillin-streptomycin. The cells covered with the hydrogel were subjected to various treatments (group 1: control group = cell culture plate, group 2: cisplatin, group 3: laser emission, group 4: Hydrogel: No drug and No laser, group 5: Hydrogel: No drug and laser, group 6: Hydrogel: drug and No laser, group 7: Hydrogel: drug and laser), while laser on groups were irradiated with laser light for 5 min. Then, the culture medium was replaced with MTT medium under 2 h of incubation. After that, precipitated formazan was dissolved in DMSO, and optical density (OD) was measured at a wavelength of 540 nm. The cell viability (%) was calculated according to Eq. 4 (Al Jahdaly et al., 2021):

$$\mathrm{Cell Viability=}\left[{\left(\mathrm{OD}\right)}_{\mathrm{test}}/{\left(\mathrm{OD}\right)}_{\mathrm{control}}\right]\mathrm{*100}$$ (4)

### 2.6 Statistical analysis

Data were collected in Microsoft Excel 2016 software, and the results were presented as the mean values ± standard deviation of at least five experiments. The Student’s *t*-test was performed to determine the statistical significance between experimental groups. *p* ≤ 0.05 was considered to be statistically significant.

## 3 Results and discussion

### 3.1 Morphological observation

**FIGURE 1:**
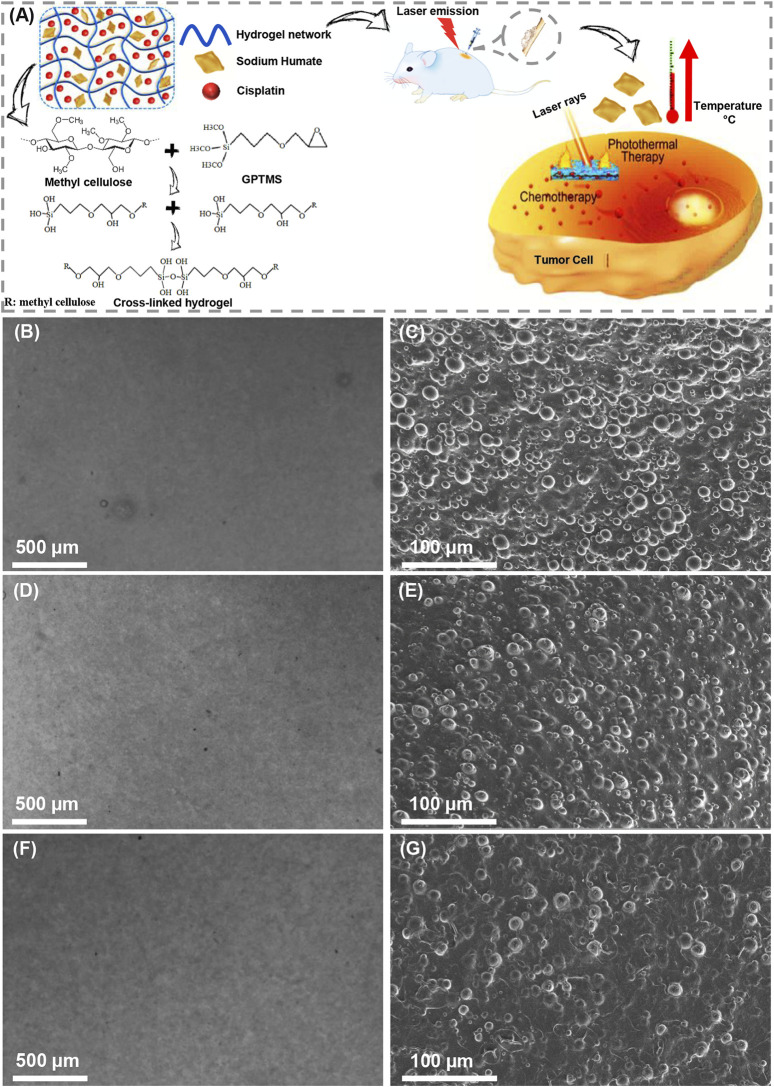
Preparation of light-assisted thermo-responsive methylcellulose-sodium humate hydrogels. **(A)** Schematic of gel formation, cross-linking reaction, and effectiveness of photothermal ablation of tumor cells. **(B,D)** and **(F)** Light microscopy images, **(C,E)** and **(G)** FE-SEM micrographs of **(B,C)** MS1, **(D,E)** MS2, and **(F,G)** MS3 hydrogels.

The properties of hydrogels are strongly influenced by the concentration of polymers, additives, and cross-linking technology (Aldana et al., 2021). In the present investigation, methylcellulose-sodium humate hydrogels were prepared, and further proposed for photothermal ablation of tumor cells and localized cisplatin delivery (Figure 1A). In this study, the concentration of methylcellulose hydrogels was adjusted by 10% (w/v), and the effects of increasing sodium humate concentrations (2.5 and 5 mg/ml) and increasing laser exposure time (5 and 10 min) were evaluated. Herein, a dark brown color hydrogel was prepared, while darkness was affected by the concentration of sodium humate. Figures 1B–G presents the light microscopy images and FESEM micrographs of MS1, MS2, and MS3 hydrogels. As displayed in light microscopy images, smooth surfaces can be observed in all hydrogels. In addition, FESEM micrographs demonstrated a higher level of uniformity in MS3 gels compared with both experimental groups. As methylcellulose exhibits a classical physical behavior, its gelation occur at elevated temperatures (Tate et al., 2001). Upon softening at ∼40°C, the hydrogel will melt at ∼50°C (Hou et al., 2018). Additionally, longer exposure times can produce more thermal energy, leading to higher gelation temperature. Accordingly, a higher uniformity of MS3 hydrogels may arise from the strong potential of sodium humate-enriched hydrogels to absorb laser light and generate more heat.

### 3.2 Chemical characterization

Methylcellulose solution in aqueous media under LCST can be hydrated through the formation of hydrogen bonds between water molecules hydroxyl functional groups; further, elevated temperature leads to hydrophobic state, breakdown of hydrogen bonds and the reversible physical gelation of this polymer (Bonetti et al., 2021; Smith et al., 2021). In this study, the gelation of methylcellulose was evaluated as a function of sodium humate concentration and laser emission time. It was found that both parameters contributed significantly to the physical gelation of methylcellulose at low temperatures by hydrating the polymeric chains. Then, elevated temperature of methylcellulose hydrogels as a function of sodium humate addition leads to water loss of macromolecules and starting self-assembles physical cross-linking and gelation of sol by destroying hydrogen bonds (Gupta et al., 2006) as well as starting intermolecular hydrophobic interactions (Morozova et al., 2019). Indeed, hydrophobic interactions between highly methylated glucose zones result in clear loose gel, which is primary step of gelation. Then, upon elevating the temperature to higher than 60°C, the phase separation occurs called turbid gel (Nasatto et al., 2015). Note that sodium and chlorine ions in could interact with methylcellulose leading to dehydration of the applied polymer. This can reduce gelation temperature and time slightly (Liu and Yao, 2015), so it is expected that gelation rapidly happens after injection and emission of laser rays causing maintained hydrogel stability and preventing from flowing in the injection site. However, the low durability of the methylcellulose-based hydrogels in body fluids increases the need for the cross-linking process (Pakulska et al., 2015). Herein, compared to physical cross-linking, chemical cross-linking reactions produces longer-lasting gels through forming intermolecular bonds and bridges between polymer chains, as well as synthesizing elastic hydrogels (Al-Sibani et al., 2015). In alkali media, the oxirane ring-opening reaction of GPTMS will be followed by deprotonation of hydroxyl groups within the chemical composition of methylcellulose. Then, the interaction of epoxy and hydroxyl functional groups leads to ether bonds formation and etherification (Schramm, 2020). Further, hydration of trimethoxy groups in the chemical composition of GPTMS leads to the formation of silanol groups, whereby Si-O-Si bonds are formed following the initiation of the acid-catalyzed reaction (Pujiasih et al., 2018) (Figure 1A). Based on the findings of De Boulle et al. (2013), hydrogels formed through cross-linking in alkaline media are more stable due to the formation of ether bonds and their resistance to enzymatic degradation.

**FIGURE 2:**
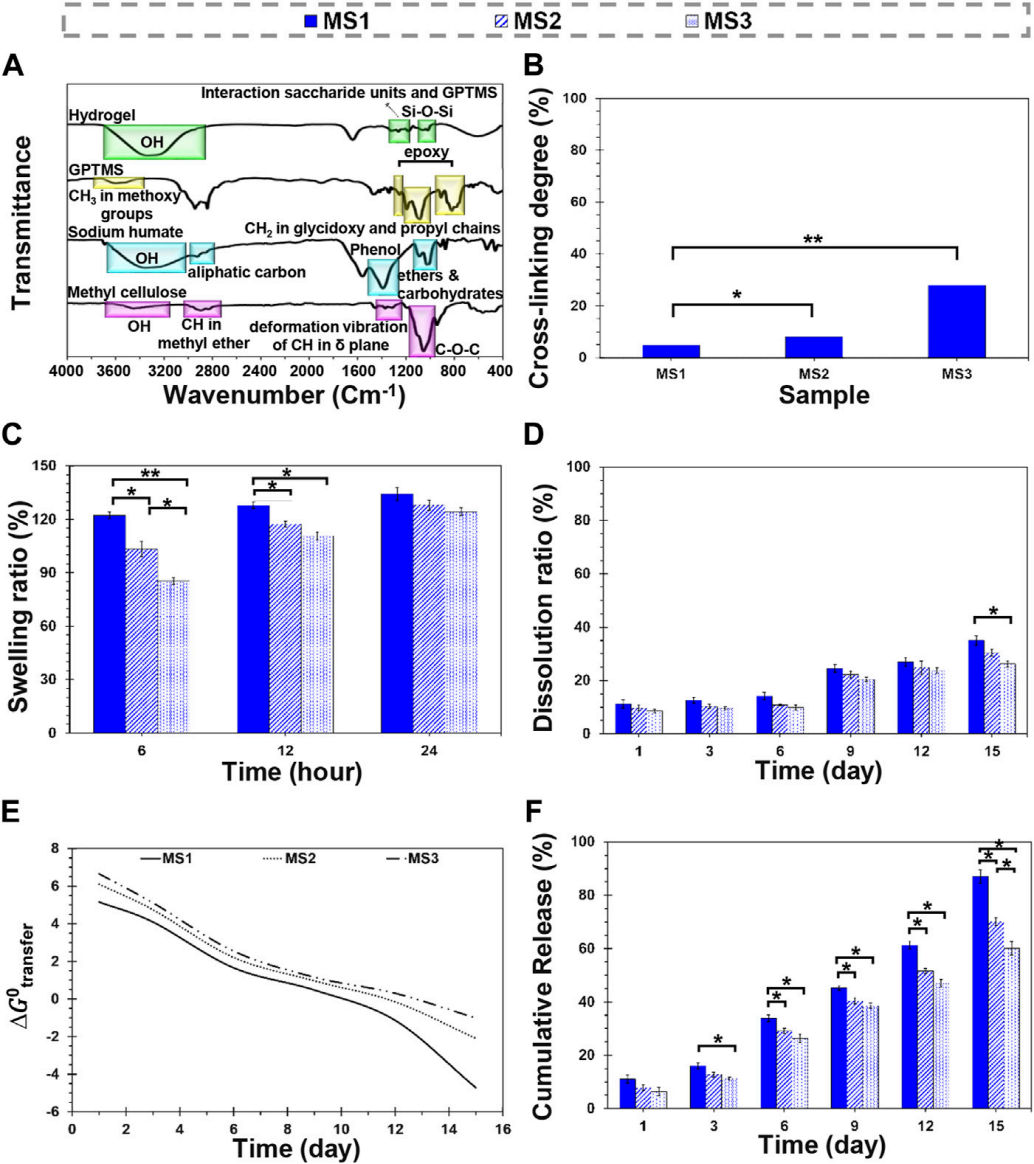
Physicochemical characterization of light-assisted thermo-responsive methylcellulose-sodium humate hydrogels. **(A)** FTIR spectra of raw materials and methylcellulose-based hydrogels, **(B)** cross-linking density of the composite hydrogels, **(C)** a 24-h swelling ratio, **(D)** a 15-day dissolution ratio of MS1, MS2, and MS3 hydrogels. **(E)** The plot of Gibbs free energy changes of cisplatin transfer and **(F)** the release profile of cisplatin from MS1, MS2, and MS3 hydrogels at 37°C ± 0.5°C. (Difference is considered to be statistically (*p* ≤ 0.05*) and very statistically (*p* ≤ 0.005**) significant).

The chemical characterization was assessed by FTIR spectroscopy (Figure 2A). The spectrum of methylcellulose determined the stretching vibration of OH groups with the peak appearing at 3,460 cm^−1^. The peaks at 2,910 and 2,837 cm^−1^ are related to the stretching vibration of CH in methyl ether. The presence of hydroxyl groups in the chemical structure of methylcellulose provides solubility in aqueous media, while the methyl groups provide crystalline phase by preventing chain-chain packing (Nasatto et al., 2015). The characteristic peaks at 1,385 cm^−1^ correspond to the deformation vibration of CH in the δ plane. C-O-C stretching related to glucosidic units was detected at 1,060 cm^−1^. The characteristic peak of sodium humate was observed at 3,200–3,400 cm^−1^ associated with the stretching of OH in carboxyl, alcohol, and phenol. The peaks appearing at 2,922 and 2,850 cm^−1^ are assigned to asymmetrical and symmetrical aliphatic carbon (CH_2_), respectively. The aromatic ethers and possibly carbohydrates were detected at 1,085 and 1,030 cm^−1^. The peaks around 1,400 cm^−1^ correspond to phenols. In the case of GPTMS, the CH_3_ stretching vibration of methoxy groups was observed at 2,944 cm^−1^. The peaks found at 1,195 and 1,081 cm^−1^ corresponded with the CH_2_ in glycidoxy and propyl chains, respectively. The peaks detected at 1,254, 910, and 859 cm^−1^ were an indication of epoxy groups. In the modified hydrogels, the disappearance of peaks at 1,254, 910, and 859 cm^−1^ revealed epoxidation reaction. Also, the peak observed at 1,337 cm^−1^ is related to the reaction between the saccharide units and GPTMS, which means cross-linking interaction. The peak at 1,020 cm^−1^ confirms the formation of Si-O-Si linkage.

Figure 2B indicates the degree of cross-linking of hydrogels. Based on obtained results, MS1, MS2, and MS3 hydrogels showed 4.86 ± 0.60, 8.07 ± 0.52, and 27.96 ± 0.74% cross-linking density, respectively. The differences between MS1 and MS2 values were statistically significant (*p* ≤ 0.05*), while MS1 and MS3 gels showed considerable significant differences (*p* ≤ 0.005**). It may result from the ascend sodium humate concentrations and increased ability of hydrogels to absorb emitted rays that improve gelation and increase viscosity. Furthermore, physical entanglement of polymeric chains and twisting into rope-like bundles can be responsible for this phenomenon (Morozova et al., 2019). A similar mechanism is also followed by increasing emission time in order to elevate the level of cross-linking. However, sodium humate’s exposure time and concentration are not the only factors associated with cross-linking degree; thus, GPTMS, a bioactive and biocompatible chemical cross-linker for amines and hydroxyls, can also be considered.

### 3.3 Water-hydrogel interactions

The swelling capacity of the hydrogels was determined by soaking the dried species in the PBS solution (Figure 2C; Supplementary Table S1). The results revealed that experimental groups swelled rapidly and absorbed PBS solution approximately between 125% and 135% of PBS within 24 h. The cross-linked network’s swelling starts and continues until the elastic and retractive forces produced by the cross-linker are balanced with the thermodynamic force of swelling (Sannino et al., 2009). It was found that the reaction of GPTMS with hydroxyl functional groups of methylcellulose terminated the formation of water-resistance bonds, which may affect the absorption rate; however, they displayed a higher absorption capacity than other methylcellulose-based gels with less than 40% swelling ratio (Liu and Yao, 2015).

In this research, MS1 hydrogel exhibited a high equilibrium swelling ratio (134.29% ± 3.71%); however, increasing light absorbance content with exposure time indicated a reduction in absorption capacity, as the MS2 and MS3 samples were only able to absorb 128.08 ± 2.82 and 124.28 ± 2.17% of PBS solution after 24 h, respectively. Although there were no significant differences in equilibrium swelling capacity, the differences between measured values were statistically significant during the initial 6 and 12 h. Higher viscosity and cross-linking density of MS3 hydrogels can be influencing factors on lower absorption capacity of this experimental group, similar to the observation of Rimdusit et al. (2008). The PBS content of hydrogels confirmed the obtained results in the PBS absorption test (Supplementary Information).

Figure 2D indicates the dissolution ratio of MS1, MS2, and MS3 hydrogels in the PBS solution (Supplementary Table S1). Accordingly, the hydrogels remained stable within a 15-day experimental period, which is similar to the gels reported by Tate et al. (2001), Liu and Yao (2015). A higher stability was observed for MS3 as a function of incubation time, which may be attributed to lower absorption capacity, higher cross-linking density, and better rheological properties; however, the statistical differences between experimental groups were not significant except on day 15 between MS1 and MS3 groups. Under *in-vivo* conditions, the fragmentation process of polymeric chains and cleavage of glycosidic linkages would initiate biodegradation, after which the fragments are metabolized to water and carbon dioxide with releasing energy, and finally eliminated by the liver and kidney (Wach et al., 2003). In this study, GPTMS was used to modify hydrogels. Hydrogels with a lower cross-linking degree are more prone to dissolution, possibly because of the density of intermolecular bonds (Wach et al., 2003). Indeed, the cross-linking process reduces the density of the sensitive groups such as hydroxyls, entraps polymeric chains, lowers the diffusion of destructive factors through a reduction in the void volume for trapping water molecules, and modulates biodegradation (Rimdusit et al., 2008). Controlled dissolution of the cross-linked methylcellulose enabled or long-term transplantation strategies due to its ability to retain water and deliver chemicals, such as cytokines or growth factors to cells (Tate et al., 2001). Note that in aqueous media, the hydrolysis of remained epoxy groups in GPTMS can be followed by production of simple diol and finally dissolution.

### 3.4 Release behavior

The changes in the structural stability of the carrier, which leads to drug release, can be described by the Gibbs free energy transfer, $\;\Delta G_{transfer}^{0}$ , using Eq. 5 (Ahuja et al., 2007):

$$\Delta G_{transfer}^{0}=-2.303RTlog\frac{C_{t}}{C_{T}}$$ (5)

Where $C_{T}$ and $C_{t}$ represent the total concentration and the concentration during the release of cisplatin, respectively. R and T are the gas constant and the temperature, respectively. Figure 2E indicates that cisplatin release from MS1 hydrogel is more spontaneous and leass controllable than MS2 and MS3 hydrogels, respectively. The $\Delta G_{transfer}^{0}$ values show that MS3 hydrogel resists structural changes for cisplatin release (Figure 2E). Thus, MS3 hydrogel demonstrates a condensed structure with higher structural strength when compared to MS2 and MS1 hydrogels. Meanwhile, MS2 hydrogels do not show a fast release of drugs with their condensed structure, as compared to MS1 and MS3 hydrogels, respectively. Hence, the difference in the chemical structure of hydrogels and formation methods leads to different thermodynamic mechanisms for the cisplatin release. Altogether, the thermodynamic standpoint suggests that MS2 hydrogel has an adaptation and more controlled release behavior under the simulation conditions.

The release profile of cisplatin from MS1, MS2, and MS3 hydrogels is shown in Figure 2F. The results show that the maximum and minimum cisplatin release has occurred from MS1 and MS3 hydrogels, respectively. The release data are fitted into mathematical models to indicate the release kinetics and mechanism (Supplementary Tables S2, S3). The kinetics results well matched the Korsmeyer-Peppas model. In addition, it shows the anomalous diffusion is the mechanism of cisplatin release from MS1, MS2, and MS3 hydrogels. The findings indicate that MS3 hydrogel resists erosion compared to MS1 and MS2 hydrogels. It suggests the diffusion mechanism is favorable for cisplatin release from the MS3 hydrogel, considering the swelling of the condensed structure once the interval has elapsed under the simulation conditions. In contrast, erosion is favorable for the MS1 and MS2 hydrogels since their structure is more accessible in the simulation conditions. The kinetics results are coherent with the thermodynamic findings since the erosion and diffusion mechanisms for cisplatin release are directly related to the structure of hydrogels and formation methods. Accordingly, considering the release kinetics and Kopcha model, MS2 hydrogel structurally adapts more with simulation conditions and has prolonged-release features.

### 3.5 Rheological properties

**FIGURE 3:**
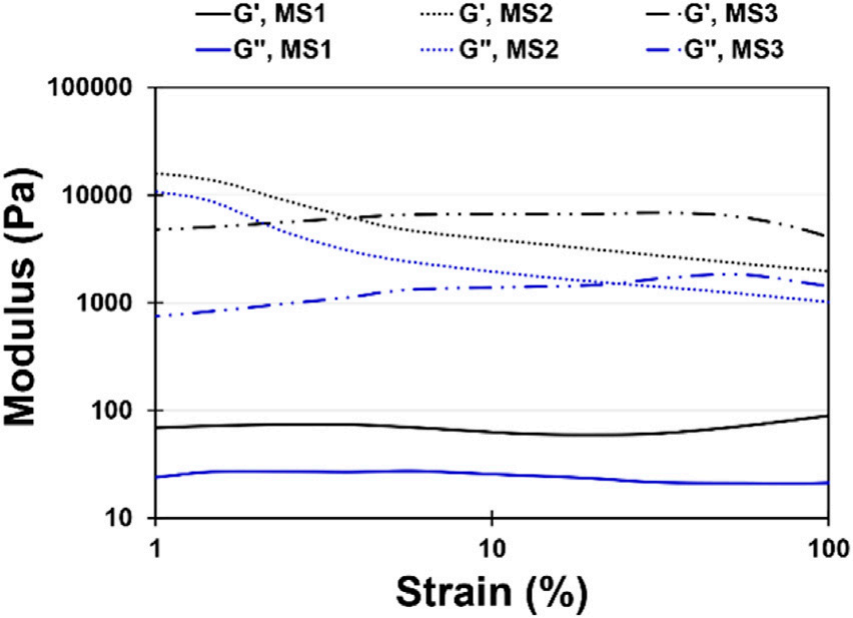
Strain sweep diagram at a constant frequency (*ω*) of 1 rad/s and strain range of 1 < *γ* < 100% for MS1, MS2, and MS3 hydrogels at constant temperature (37°C ± 0.5°C).

**FIGURE 4:**
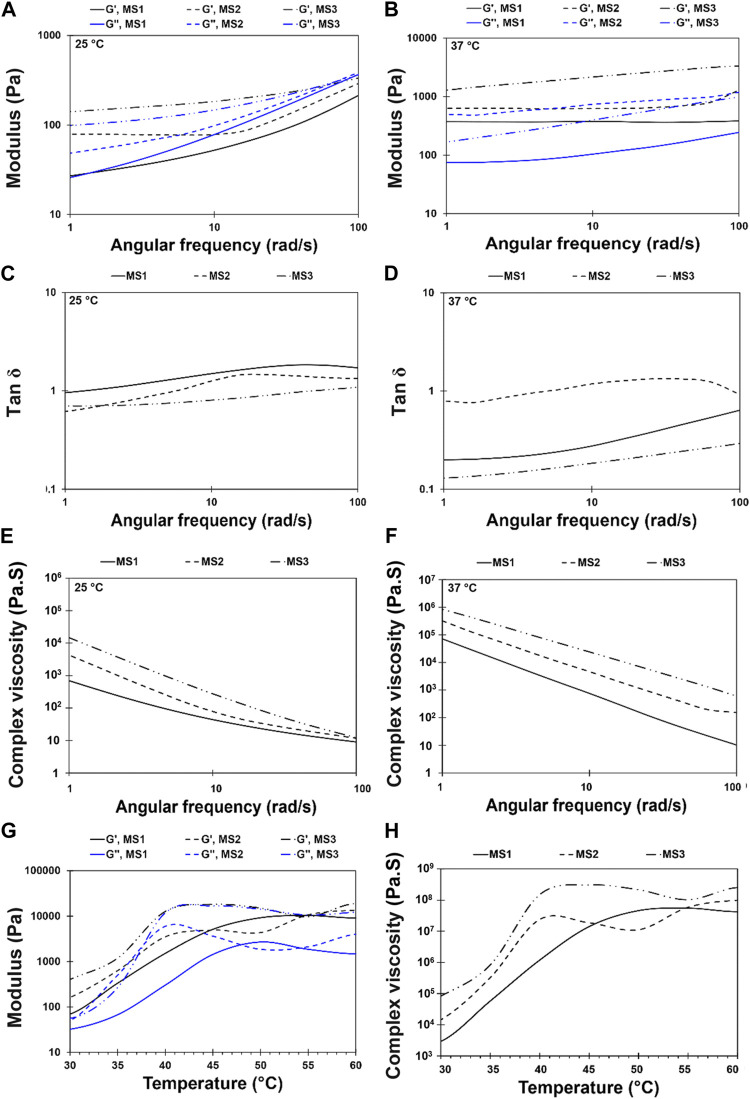
Rheological behavior of light-assisted thermo-responsive methylcellulose-sodium humate hydrogels. Frequency sweep diagram at an angular frequency of 1 < *ω* < 100 rad/s and 10% strain for MS1, MS2, and MS3 hydrogels at temperature **(A,C,E)** 25 ± 0.5°C and **(B,D,F)** 37 ± 0.5°C [**(A,B)** Storage and loss modulus, **(C,D)** Tan δ, and **(E,F)** complex viscosity]. **(G,H)** Temperature sweep diagram at a constant angular frequency of 1 rad/s and temperature range of 30 to 60°C at a heating rate of 1°C/min for MS1, MS2, and MS3 hydrogels [**(G)** Storage and loss modulus, and **(H)** complex viscosity].

Injectable hydrogels need to possess viscoelastic properties so that a viscose behavior is required during injection, since shear stress is applied, while the injected gels should be elastic enough to maintain their shape at the defect site (Ghorbani et al., 2020). In this research, a strain sweep was performed to determine the mechanical stability of the hydrogels and to identify the linear region (Figure 3). The frequency sweep test was carried out at both room and body temperature to understand the frequency-dependent viscoelastic behavior of MS1, MS2, and MS3 hydrogels. Figures 4A–F demonstrates the results of frequency sweep analysis at constant strain and ambient temperature (A, C, and E) and body temperature (B, D, and F). It was confirmed that hydrogels are capable of performing viscoelastic functions. A viscose behavior was observed at lower frequencies, while elastic behavior was observed at higher frequencies related to short-duration forces. Additionally, higher values of storage modulus than loss modulus are indicative of gel formation, as reported in similar literature (Liu and Yao, 2015). The results indicated that the samples with a higher concentration of light absorbance and laser light emission displayed greater storage modulus, possibly as a result of the higher viscosity of the MS3 hydrogels. According to the results of complex viscosity analysis as a function of frequency enhancement, the MS3 samples had a higher viscosity than the other groups. Further, reduction in viscosity of all experimental groups as a function of frequency is indicative of non-Newton fluid behavior of the hydrogels associated with chain-chain interactions, similar to other published research (Nasatto et al., 2015). The hydrogels presented about 10 times greater storage modulus at body temperature than at ambient ones. Additionally, the gels analyzed at body temperature showed a lower dependence of storage modulus on ascending frequencies as a result of broader linear viscoelastic regions. An increase in viscosity was also observed after enhancing the experiment temperature. These results indicate increased rigidity and durability of hydrogels, which prevent rapid deformation of the hydrogels. Herein, approximately lower levels of tanσ in MS3 gels proved the predominance of elastic behavior to viscose one, especially at body temperature.

Light-assisted thermo-responsive behavior of methylcellulose-based hydrogels has been presented in Figures 4G,H. Accordingly, the hydrogels indicated light-assisted thermo-responsive gelation within the temperature range of 30°C–60°C. The gelation temperature of MS2 and MS3 gels was determined based on the cross-point of storage and loss moduli, as shown in Figure 4. Nevertheless, there was no point for MS1 hydrogel, and loss modulus was smaller than the storage one, showing a typical solid-state behavior. However, a reduction of sol-gel transition temperature from ∼50°C to 40°C and 43°C, respectively, in MS2 and MS3 hydrogels, demonstrated the role of PBS in the dehydration process and fall of transition temperature (Xu et al., 2004). Increasing both storage and loss moduli as a function of temperature elevation, which indicates the sol to gel transition, enhances the stability of methylcellulose-based hydrogel. As other studies showed, gelation of sol results in physical entanglement of polymeric chains and twisting into rope-like bundles (Morozova et al., 2019); consequently, the density of cross-linking and mechanical performance can be improved. Herein, MS3 sample showed higher storage modulus and viscosity compared with other groups. Further, the semi-dependent behavior of storage modulus on elevated temperatures can be observed in MS3 hydrogels after gelation. Furthermore, the hydrogels demonstrated a low viscosity at lower temperatures, making them suitable for injection into defects. It is expected that after injection of gels and emission of laser light which is followed by raising the temperature, the gelation would occur and reduce gel movement owing to increasing viscosity as a function of elevated temperature (Figure 4H); the same results were obtained by Tate et al. (2001).

### 3.6 The results of hydrogel interaction with the model proteins

**FIGURE 5:**
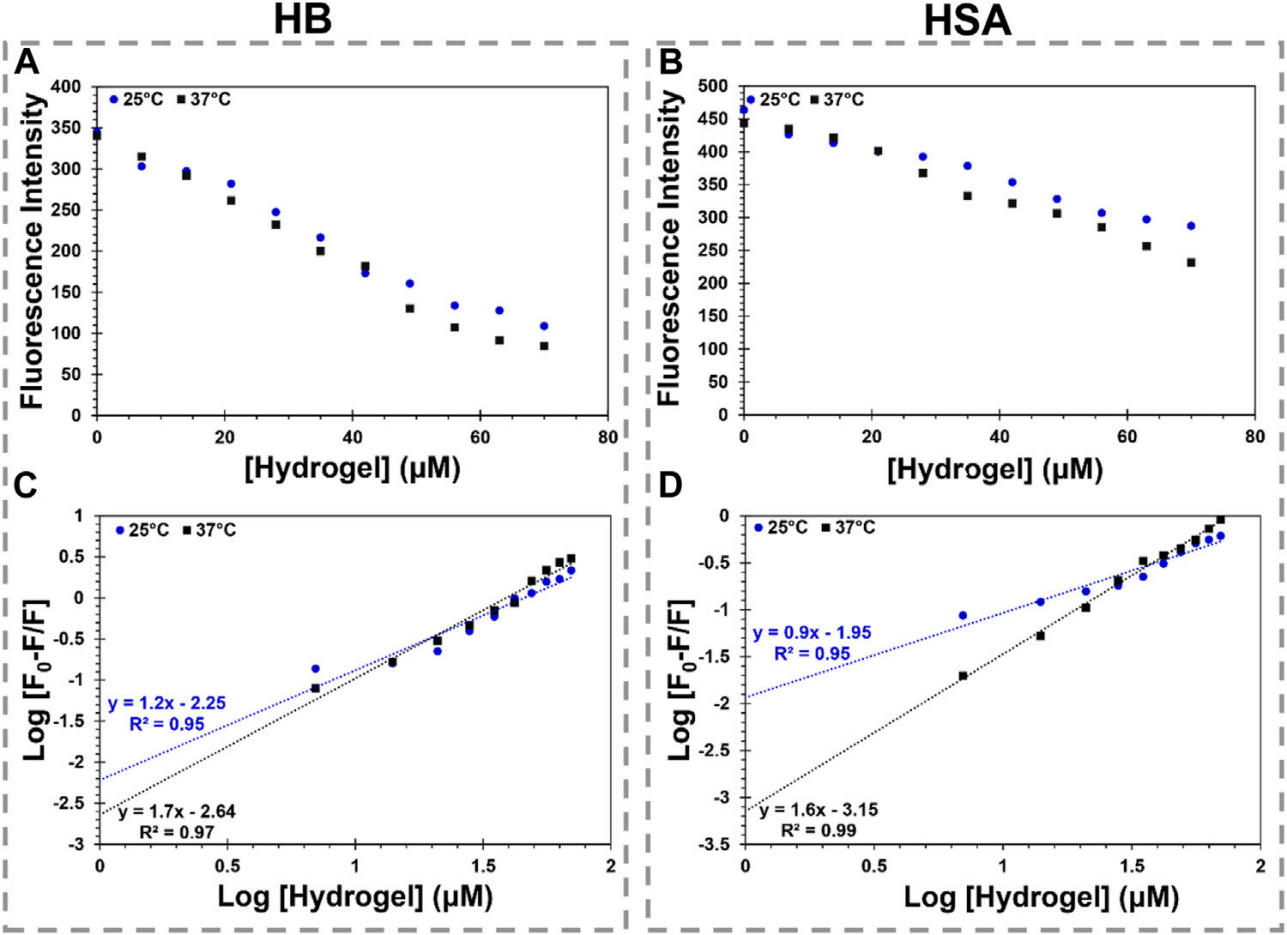
MS2 hydrogel interaction with HB and HSA. **(A,B)** show the changes in maximum fluorescence intensity of HB and HSA by MS2 hydrogel (0–70 µM), respectively. **(C,D)** show the logarithmic graph of HB and HSA binding to MS2 hydrogel at various temperatures, respectively.

The maximum fluorescence intensity of HSA and HB regularly diminished with each addition of MS2 hydrogel at 25°C and 37°C [Figures 5A,B, Supplementary Figure S2 (Supplementary Information)]. In addition, Figures 5A,B illustrates a direct interdependence between the fluorescence intensity quenching of the model proteins and the hydrogel concentration. The quenching data indicate a complex between the MS2 hydrogel and the model proteins (Supplementary Information, Supplementary Figure S3; Supplementary Table S3). To assess the complex formation and binding parameters, the fluorescence quenching data are fitted to Eq. 6.

$$log\left[\frac{F_{0}-F}{F}\right]=logK_{a}+nlog\left[Q\right]$$ (6)

Where *K*_*a*_ and *n* represent the association binding constant and the number of the binding sites, respectively (Shafaei et al., 2017). The plots of hydrogel binding to the model proteins are shown in Figures 5C,D. The obtained values of the binding parameters are also reported in Table 2. At 25°C, the number of the binding sites is close to one, revealing the one-by-one stoichiometry of the interaction between the hydrogel and the model proteins. On the other hand, at 37°C, the number of the binding sites grows to approximately two, indicating that the model proteins’ conformation is altered upon interaction with the hydrogel as the temperature rises. The calculated binding constant for the interaction of the model proteins with the hydrogel diminished with raising temperature. It means the binding process in all cases is exothermic.

**TABLE 2:** Binding and thermodynamic parameters for the hydrogel interaction with the model proteins.

Protein	Temperature (°C)	n	K_a_ (×10^6^M^−1^)	ΔG^0^ (kJ/mol)	ΔH^0^ (kJ/mol)	ΔS^0^ (J/mol. K)
HSA	25	0.9	0.0112	−23.12	−193.29	−570.77
	37	1.6	0.0007	−16.27		
Hb	25	1.2	0.0056	−21.4	−76.71	−185.5
	37	1.7	0.0023	−19.17		

The binding constant is dramatically reduced, and a weak binding is formed with temperature rise in accordance with the Stern–Volmer plots, suggesting the complexes are driven into instability. According to the binding constant dependency on the temperature, the van’t Hoff equation (Eq. 7) calculates the changes in the enthalpy (ΔH^0^) and entropy (ΔS^0^) of the interaction. Then, the Gibbs free energy (ΔG^0^) of the reaction is obtained using Eq. 8.

$$\ln K_{a}\mathrm{=-}\frac{\Delta H^{0}}{\mathrm{RT}}+\frac{\Delta S^{0}}{R}$$ (7)

$$\Delta G^{0}=\Delta H^{0}-T\Delta S^{0}=-RTlnK_{a}$$ (8)

Where R is the gas constant and T denotes the absolute temperature (Ke et al., 2021). The obtained thermodynamics parameters are summarized in Table 2. As seen, both ΔH^0^ and ΔS^0^ are negative, indicating the binding process of the model proteins to the hydrogel is governed by the hydrogen bond and van der Waals forces. In addition, in all cases, the negative sign of ΔG^0^ indicates the binding process is spontaneous. The findings suggest that the stability of the HSA-hydrogel complex and the HB-hydrogel complex is different due to the hydrogel’s chemical structure features and the surface properties of the model proteins. Although the hydrogel-HSA complex is more stable than the hydrogel-HB complex at 25°C, the complex stability changes with temperature elevation. It suggests that the conformational changes in the model proteins have a significant impact on complex stability. Altogether, the interaction study reveals a significant finding regarding the proteins-hydrogel complexation in terms of biocompatibility.

The complex instability of the model proteins-hydrogel, considering the effect of the laser irradiation, enables the practical application of hydrogel for drug delivery. Due to the hydrogel’s favorable performance in relation to laser radiation, elevation of temperature by laser light is thought to control the hydrogel’s function following its complexation with proteins in the body. Meanwhile, the complex formation with the proteins as an extra layer by a reversible reaction can enhance the half-life of encapsulated drug and prevent a rapid release of drug in the body. Finally, the system with augmentation of temperature by laser irradiation loses the extra layer of protein first, after which the drug is released at the target site.

### 3.7 Cell viability

**FIGURE 6:**
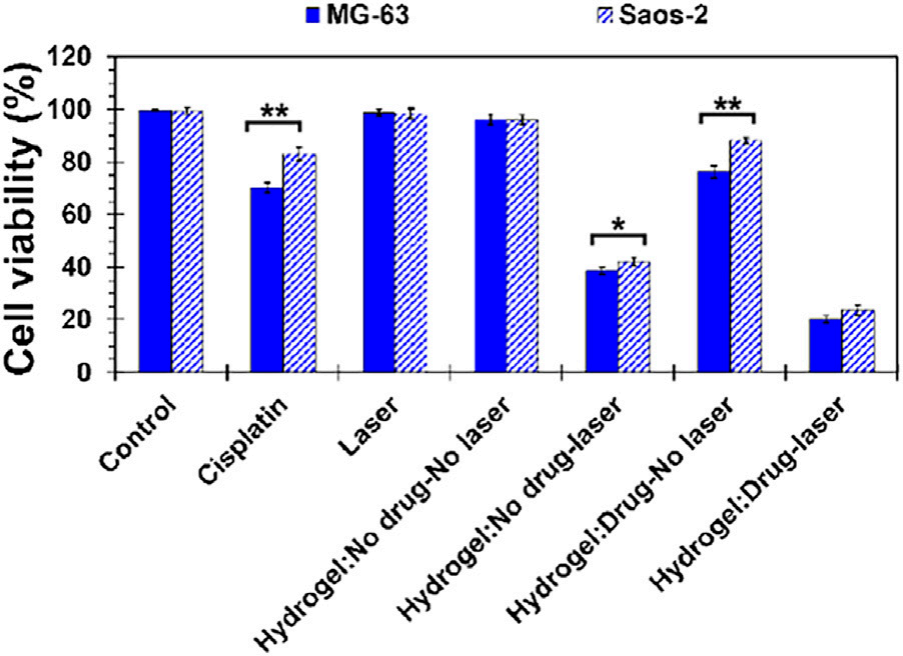
Behavior of MS2 hydrogels against osteosarcoma cell lines (Saos-2 and MG-63) by MTT assay after 48 h culturing the cells. [Difference is considered to be statistically (*p* ≤ 0.05*) and very statistically (*p* ≤ 0.005**) significant].

Biomedical application of prepared MS2 hydrogels and primary effectiveness against cancer cells were evaluated by MTT assay, as depicted in Figure 6. Here, osteosarcoma cell lines (Saos-2 and MG-63) were treated for 48 h with different experimental groups to determine the synergistic effect of thermo-responsive hydrogels and cisplatin. According to the observation, there was no considerable toxicity in the control group, laser emission, and cisplatin-free hydrogels (laser off). Mild toxicity was detected in free cisplatin and cisplatin-loaded hydrogels (laser off), which was attributed to the chemotherapeutic effects of cisplatin. The possible mechanism for such phenomena may be the sensitivity of the osteosarcoma cells to cisplatin and the reduction in the possibility of drug resistance (Jhaveri et al., 2014). However, photothermal ablation of tumor cells was achieved in cisplatin-free hydrogels when hydrogels were subjected to laser light emission. In this condition, cell viability was 38.63 ± 1.37 and 42.16 ± 1.50% for MG-63 and Saos-2 cells, respectively. Nonetheless, the number of dead cells was intensified when chemotherapy agent and photothermal stimuli were introduced simultaneously to the culture system. In this case, 20.12 ± 1.18 and 23.70 ± 1.97 of MG-63 and Saos-2 cells, respectively, were viable, indicating the synergistic cooperation of cisplatin and laser light to suppress tumor cells under chemo-photothermal therapeutics *in-vitro*.

## 4 Conclusion

Ethylcellulose hydrogel was chemically modified with silane coupling agent and combined with sodium humate and cisplatin in order to produce an injectable light-assisted thermo-responsive hydrogel with drug release capabilities. When sodium humate is exposed to laser light, it undergoes a temperature increase, resulting in a transition of methylcellulose from a sol to a gel. The result is that less resistance will be observed during injection time, and gelation will occur quickly after injection in addition to the emission of laser rays that will prevent products from flowing in the injection site. Further, the temperature-dependent nature of the gelation process was confirmed by rheological behavior evaluation (temperature sweep mode). The light to thermal conversion resulted in the stabilization of the hydrogels, which in turn resulted in improved control over the release of the chemotherapy agents. At the same time, it generated thermal energy which may contribute to the ablation of tumor cells by photothermal means. Further, we observed that the external stimuli factor and light-absorbance concentration are essential factors in determining the rate of absorption, durability and long-term stability of injectable hydrogels, storage modules, and viscosity, as well as the sustained release rate of cisplatin, which may influence the simultaneous and synergistic effects of chemo-photothermal therapy. Due to the structural characteristics of the hydrogel and the synthesis method employed, the release of cisplatin followed a non-Fickian mechanism with the predominant contribution of erosion under the simulation conditions. The findings suggest that protein-hydrogel complexation boosts the performance of hydrogel in the delivery system. The synergistic effect between laser emission and cisplatin release on reducing the number of viable osteosarcoma cell lines suggests the possibility of tumor ablation. The light responsive methylcellulose-sodium humate hydrogels, which are a cisplatin carrier, have provided initial indications for further preclinical and clinical investigation of osteosarcoma treatment.

## Data Availability

The original contributions presented in the study are included in the article/Supplementary Material, further inquiries can be directed to the corresponding author.

## References

[B1] Abed Z. Khoei S. Ghalandari B. Beik J. Shakeri-Zadeh A. Ghaznavi H. (2018). The measurement and mathematical analysis of 5-fu release from magnetic polymeric nanocapsules, following the application of ultrasound. Anticancer. Agents Med. Chem. 18, 438–449. 10.2174/1871520617666170921124951 28933262

[B2] Ahuja N. Katare O. P. Singh B. (2007). Studies on dissolution enhancement and mathematical modeling of drug release of a poorly water-soluble drug using water-soluble carriers. Eur. J. Pharm. Biopharm. 65, 26–38. 10.1016/j.ejpb.2006.07.007 16962750

[B3] Al Jahdaly B. A. Al-Radadi N. S. Eldin G. M. G. Almahri A. Ahmed M. K. Shoueir K. (2021). Selenium nanoparticles synthesized using an eco-friendly method: Dye decolorization from aqueous solutions, cell viability, antioxidant, and antibacterial effectiveness. J. Mater. Res. Technol. 11, 85–97. 10.1016/j.jmrt.2020.12.098

[B4] Al-Sibani M. Al-Harrasi A. Neubert R. H. H. (2015). Evaluation of *in-vitro* degradation rate of hyaluronic acid-based hydrogel cross-linked with 1, 4-butanediol diglycidyl ether (BDDE) using RP-HPLC and UV–Vis spectroscopy. J. Drug Deliv. Sci. Technol. 29, 24–30. 10.1016/j.jddst.2015.05.013

[B5] Aldana A. A. Houben S. Moroni L. Baker M. B. Pitet L. M. (2021). Trends in double networks as bioprintable and injectable hydrogel scaffolds for tissue regeneration. ACS Biomater. Sci. Eng. 7, 4077–4101. 10.1021/acsbiomaterials.0c01749 33606938

[B6] Barkoula N. M. Alcock B. Cabrera N. O. Peijs T. (2008). Fatigue properties of highly oriented polypropylene tapes and all-polypropylene composites. Polym. Polym. Compos. 16, 101–113. 10.1177/096739110801600203

[B7] Bonetti L. De Nardo L. Farè S. (2021). Thermo-responsive methylcellulose hydrogels: From design to applications as smart biomaterials. Tissue Eng. Part B Rev. 27, 486–513. 10.1089/ten.teb.2020.0202 33115329

[B8] Coughlin M. L. Liberman L. Ertem S. P. Edmund J. Bates F. S. Lodge T. P. (2021). Methyl cellulose solutions and gels: Fibril formation and gelation properties. Prog. Polym. Sci. 112, 101324. 10.1016/j.progpolymsci.2020.101324

[B9] De Boulle K. Glogau R. Kono T. Nathan M. Tezel A. Roca-Martinez J.-X. (2013). A review of the metabolism of 1, 4-butanediol diglycidyl ether-cross-linked hyaluronic acid dermal fillers. Dermatol. Surg. 39, 1758–1766. 10.1111/dsu.12301 23941624 PMC4264939

[B10] Ghorbani F. Zamanian A. Behnamghader A. Daliri Joupari M. (2020). Bioactive and biostable hyaluronic acid-pullulan dermal hydrogels incorporated with biomimetic hydroxyapatite spheres. Mater. Sci. Eng. C 112, 110906. 10.1016/j.msec.2020.110906 32409060

[B11] Ghorbani F. Zamanian A. Shams A. Shamoosi A. Aidun A. (2019). Fabrication and characterisation of super-paramagnetic responsive PLGA–gelatine–magnetite scaffolds with the unidirectional porous structure: A physicochemical, mechanical, and *in vitro* evaluation. IET Nanobiotechnol. 13, 860–867. 10.1049/iet-nbt.2018.5305 31625528 PMC8676357

[B12] Gupta D. Tator C. H. Shoichet M. S. (2006). Fast-gelling injectable blend of hyaluronan and methylcellulose for intrathecal, localized delivery to the injured spinal cord. Biomaterials 27, 2370–2379. 10.1016/j.biomaterials.2005.11.015 16325904

[B13] Hou G. Qian J. Guo M. Xu W. Wang J. Wang Y. (2022). Hydrazided hyaluronan/cisplatin/indocyanine green coordination nanoprodrug for photodynamic chemotherapy in liver cancer. Carbohydr. Polym. 276, 118810. 10.1016/j.carbpol.2021.118810 34823812

[B14] Hou M. Yang R. Zhang L. Zhang L. Liu G. Xu Z. (2018). Injectable and natural humic acid/agarose hybrid hydrogel for localized light-driven photothermal ablation and chemotherapy of cancer. ACS Biomater. Sci. Eng. 4, 4266–4277. 10.1021/acsbiomaterials.8b01147 33418824

[B15] Jhaveri A. Deshpande P. Torchilin V. (2014). Stimuli-sensitive nanopreparations for combination cancer therapy. J. Control. Release 190, 352–370. 10.1016/j.jconrel.2014.05.002 24818767

[B16] Jo Y.-J. Gulfam M. Jo S.-H. Gal Y.-S. Oh C.-W. Park S.-H. (2022). Multi-stimuli responsive hydrogels derived from hyaluronic acid for cancer therapy application. Carbohydr. Polym. 286, 119303. 10.1016/j.carbpol.2022.119303 35337532

[B17] Ke Y. Huang S. Ghalandari B. Li S. Warden A. R. Dang J. (2021). Hairpin‐spacer crRNA‐enhanced CRISPR/Cas13a system promotes the specificity of single nucleotide polymorphism (SNP) identification. Adv. Sci. 8, 2003611. 10.1002/advs.202003611 PMC7967054 33747742

[B18] Kim Y. Hu Y. Jeong J. Jung S. (2022). Injectable, self-healable and adhesive hydrogels using oxidized Succinoglycan/chitosan for pH-responsive drug delivery. Carbohydr. Polym. 284, 119195. 10.1016/j.carbpol.2022.119195 35287911

[B19] Liu Z. Yao P. (2015). Injectable thermo-responsive hydrogel composed of xanthan gum and methylcellulose double networks with shear-thinning property. Carbohydr. Polym. 132, 490–498. 10.1016/j.carbpol.2015.06.013 26256374

[B20] Luetke A. Meyers P. A. Lewis I. Juergens H. (2014). Osteosarcoma treatment – where do we stand? A state of the art review. Cancer Treat. Rev. 40, 523–532. 10.1016/j.ctrv.2013.11.006 24345772

[B21] Ma H. He C. Cheng Y. Yang Z. Zang J. Liu J. (2015). Localized Co-delivery of doxorubicin, cisplatin, and methotrexate by thermosensitive hydrogels for enhanced osteosarcoma treatment. ACS Appl. Mat. Interfaces 7, 27040–27048. 10.1021/acsami.5b09112 26575336

[B22] Morozova S. Coughlin M. L. Early J. T. Ertem S. P. Reineke T. M. Bates F. S. (2019). Properties of chemically cross-linked methylcellulose gels. Macromolecules 52, 7740–7748. 10.1021/acs.macromol.9b01401

[B23] Nasatto P. L. Pignon F. Silveira J. L. M. Duarte M. E. R. Noseda M. D. Rinaudo M. (2015). Methylcellulose, a cellulose derivative with original physical properties and extended applications. Polym. (Basel) 7, 777–803. 10.3390/polym7050777

[B24] Pakulska M. M. Vulic K. Tam R. Y. Shoichet M. S. (2015). Hybrid cross-linked methylcellulose hydrogel: A predictable and tunable platform for local drug delivery. Adv. Mat. 27, 5002–5008. 10.1002/adma.201502767 26184559

[B25] Pedige M. P. H. Asoh T.-A. Hsu Y.-I. Uyama H. (2022). Stimuli-responsive composite hydrogels with three-dimensional stability prepared using oxidized cellulose nanofibers and chitosan. Carbohydr. Polym. 278, 118907. 10.1016/j.carbpol.2021.118907 34973728

[B26] Pujiasih S. KurniaMasykur A. Kusumaningsih T. Saputra O. A. (2018). Silylation and characterization of microcrystalline cellulose isolated from Indonesian native oil palm empty fruit bunch. Carbohydr. Polym. 184, 74–81. 10.1016/j.carbpol.2017.12.060 29352945

[B27] Qin X. Xu Y. Zhou X. Gong T. Zhang Z.-R. Fu Y. (2021). An injectable micelle-hydrogel hybrid for localized and prolonged drug delivery in the management of renal fibrosis. Acta Pharm. Sin. B 11, 835–847. 10.1016/j.apsb.2020.10.016 33777685 PMC7982499

[B28] Qin Z. Wang Y. Randrianalisoa J. Raeesi V. Chan W. C. W. Lipiński W. (2016). Quantitative comparison of photothermal heat generation between gold nanospheres and nanorods. Sci. Rep. 6, 29836. 10.1038/srep29836 27445172 PMC4956767

[B29] Rimdusit S. Jingjid S. Damrongsakkul S. Tiptipakorn S. Takeichi T. (2008). Biodegradability and property characterizations of Methyl Cellulose: Effect of nanocompositing and chemical cross-linking. Carbohydr. Polym. 72, 444–455. 10.1016/j.carbpol.2007.09.007

[B30] Sannino A. Demitri C. Madaghiele M. (2009). Biodegradable cellulose-based hydrogels: Design and applications. Mater. (Basel) 2, 353–373. 10.3390/ma2020353

[B31] Schramm C. (2020). High temperature ATR-FTIR characterization of the interaction of polycarboxylic acids and organotrialkoxysilanes with cellulosic material. Spectrochimica Acta Part A Mol. Biomol. Spectrosc. 243, 118815. 10.1016/j.saa.2020.118815 32861204

[B32] Shafaei Z. Abazari O. Divsalar A. Ghalandari B. Poursoleiman A. Saboury A. A. (2017). Effect of a synthesized amyl-Glycine1, 10-phenanthroline platinum nitrate on structure and stability of human blood carrier protein, Albumin: Spectroscopic and modeling approaches. J. Fluoresc. 27, 1829–1838. 10.1007/s10895-017-2120-4 28555407

[B33] Smith K. A. Dang M. Baker A. E. G. Fuehrmann T. Fokina A. Shoichet M. S. (2021). Synthesis of an enzyme-mediated reversible cross-linked hydrogel for cell culture. Biomacromolecules 22, 5118–5127. 10.1021/acs.biomac.1c01086 34752066

[B34] Tang H.-X. Liu C.-G. Zhang J.-T. Zheng X. Yang D.-Y. Kankala R. K. (2020). Biodegradable quantum composites for synergistic photothermal therapy and copper-enhanced chemotherapy. ACS Appl. Mat. Interfaces 12, 47289–47298. 10.1021/acsami.0c14636 32975929

[B35] Tate M. C. Shear D. A. Hoffman S. W. Stein D. G. LaPlaca M. C. (2001). Biocompatibility of methylcellulose-based constructs designed for intracerebral gelation following experimental traumatic brain injury. Biomaterials 22, 1113–1123. 10.1016/S0142-9612(00)00348-3 11352091

[B36] Wach R. A. Mitomo H. Nagasawa N. Yoshii F. (2003). Radiation cross-linking of methylcellulose and hydroxyethylcellulose in concentrated aqueous solutions. Nucl. Instrum. Methods Phys. Res. Sect. B Beam Interact. Mater. Atoms 211, 533–544. 10.1016/S0168-583X(03)01513-1

[B37] Xu Y. Wang C. Tam K. C. Li L. (2004). Salt-assisted and salt-suppressed Sol−Gel transitions of methylcellulose in water. Langmuir 20, 646–652. 10.1021/la0356295 15773087

[B38] Xu Z. Yang D. Long T. Yuan L. Qiu S. Li D. (2022). pH-Sensitive nanoparticles based on amphiphilic imidazole/cholesterol modified hydroxyethyl starch for tumor chemotherapy. Carbohydr. Polym. 277, 118827. 10.1016/j.carbpol.2021.118827 34893244

[B39] Yu N. Hu Y. Wang X. Liu G. Wang Z. Liu Z. (2017). Dynamically tuning near-infrared-induced photothermal performances of TiO 2 nanocrystals by Nb doping for imaging-guided photothermal therapy of tumors. Nanoscale 9, 9148–9159. 10.1039/C7NR02180A 28650058

[B40] Yu N. Li J. Wang Z. Yang S. Liu Z. Wang Y. (2018). Blue Te nanoneedles with strong NIR photothermal and laser-enhanced anticancer effects as "All-in-One" nanoagents for synergistic thermo-chemotherapy of tumors. Adv. Healthc. Mat. 7, 1800643. 10.1002/adhm.201800643 30160820

[B41] Yu N. Qiu P. Ren Q. Wen M. Geng P. Macharia D. K. (2021a). Transforming a sword into a knife: Persistent phototoxicity inhibition and alternative therapeutical activation of highly-photosensitive phytochlorin. ACS Nano 15, 19793–19805. 10.1021/acsnano.1c07241 34851096

[B42] Yu N. Tu W. Qiu P. Ren Q. Chen X. Zhu M. (2022). Full-route advances via biomimetic and biodegradable ultrasmall-in-nano architectures with radiation-photo synergy. Nano Today 43, 101427. 10.1016/j.nantod.2022.101427

[B43] Yu Y. Cheng Y. Tong J. Zhang L. Wei Y. Tian M. (2021b). Recent advances in thermo-sensitive hydrogels for drug delivery. J. Mat. Chem. B 9, 2979–2992. 10.1039/D0TB02877K 33885662

[B44] Zhang W. Ling C. Li X. Sheng R. Liu H. Zhang A. (2020). Cell-free biomimetic scaffold with cartilage extracellular matrix-like architectures for *in situ* inductive regeneration of osteochondral defects. ACS Biomater. Sci. Eng. 6, 6917–6925. 10.1021/acsbiomaterials.0c01276 33320617

[B45] Zhu X. Guan B. Sun Z. Tian X. Li X. (2021). Fabrication of an injectable hydrogel with inherent photothermal effects from tannic acid for synergistic photothermal-chemotherapy. J. Mat. Chem. B 9, 6084–6091. 10.1039/D1TB01057C 34286812

