# Supplementary material for: Injectable light-assisted thermo-responsive methylcellulose-sodium humate hydrogel proposed for photothermal ablation and localized delivery of cisplatin
Source: Front Bioeng Biotechnol. 2022 Aug 8;10:967438. doi: 10.3389/fbioe.2022.967438 (PMC9395131; doi:10.3389/fbioe.2022.967438)
Supplement: Supplementary file 1 [file DataSheet1.pdf]

Injectable light-assisted thermo-responsive methylcellulose-sodium humate hydrogel proposed for photothermal ablation and localized delivery of cisplatin

Farnaz Ghorbani [a1#], Behafarid Ghalandari [b1], Zichen Liu [a,c], Dejian Li [a], Baoqing Yu [d*]

[a] Department of Orthopedics, Shanghai Pudong Hospital, Fudan University Pudong Medical Center, Shanghai, China.

[b] State Key Laboratory of Oncogenes and Related Genes, Institute for Personalized Medicine, School of Biomedical Engineering, Shanghai Jiao Tong University, Shanghai, China.

[c] School of Materials Science and Engineering, University of Shanghai for Science and Technology, Shanghai, China.

[d] Department of Orthopedics, Shanghai Pudong New Area People's Hospital, Shanghai, China.

*Corresponding author: Prof. Baoqing Yu; Tel: (+86)15901881828; Fax: (+86)21-58022995;

E-mail: doctorybq@163.com

# Current affiliation: Institute of Biomaterials, University of Erlangen-Nuremberg, Cauerstr. 6, 91058 Erlangen, Germany

1 These authors contributed to this work equally

Experimental:

The potential of hydrogels to interact with water molecules was evaluated by water content measurement. So, the dried hydrogels were weighted (Wi), and after incubation of samples in 30 ml of the PBS solution at 37±0.5 °C, the wet weight (Ww) was measured at a pre-determined time (6, 12, and 24 hours). Finally, the water content of hydrogels was then calculated using Eq. 1 [1]:

$$\text{Water content (\%)} = [\text{swelling} / (\text{swelling}+1)]*100 \quad (1)$$

Results:

- *Water content*

As indicated in Fig. S1, MS1 hydrogels show low storage modulus compared with other experimental groups. Herein, MS2 and MS3 gels indicated about 80 times higher stability than MS1. Although MS2 hydrogels indicated higher storage modulus in low strain than MS3, a larger linear viscoelastic region in MS3 hydrogels presented higher structural integrity. The stable performance of MS3 hydrogels with high storage modulus and stiffness may arise from binding energy, as was observed in other literature [2–5]. Besides, the higher potential of MS3 hydrogels to produce thermal energy (affected by the concentration of sodium humate and exposure time) and promoting cross-linking should not be ignored.

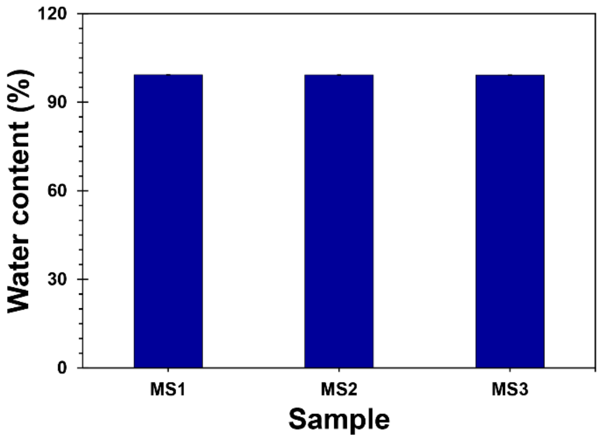

(Fig. S1) Water content of MS1, MS2, and MS3 hydrogels.

Table S1: Physical properties of synthesized hydrogels.

Sample code	A 24-hour swelling ratio (%)	A 24-hour PBS content (%)	A 15-day biodegradation ratio (%)
**MS1**	134.29±3.71	99.26±0.02	35.08±1.72
**MS2**	128.08±2.82	99.22±0.01	30.31±1.26
**MS3**	124.28±2.17	99.19±0.01	26.25±1.12

- *Release*

The release data are fitted in mathematical models to indicate the release kinetics and mechanism. According to the correlation coefficient (R^2) value, the best release model is selected for cisplatin release from MS1, MS2, and MS3 hydrogel. The mathematical kinetics models of zero-order, first-order [6], Weibull [7], Higuchi [8], Hixson–Crowell [9], and Korsmeyer–Peppas [10]. (Eqs. 1-6) are used for the release data fitting:

Zero-order: $$M_t = M_0 + k_0 t \quad (1)$$

First-order: $$\log M_t = \log M_0 + \frac{k_1 t}{2.303} \quad (2)$$

Weibull: $$\frac{M_t}{M_\infty} = 1 - \exp(-a\, t^b) \quad (3)$$

Higuchi: $$M_t = k_H \sqrt{t} \quad (4)$$

Hixson-Crowell: $$(w_0)^{1/3} - (w_t)^{1/3} = k_{HC} t \quad (5)$$

Korsmeyer-Peppas: $$\frac{M_t}{M_\infty} = k_{KP} t^n \quad (6)$$

Where M_0, M_t, and M_∞ represent the drug released amount at time zero, t, and infinity, respectively; t represents release time. In the Hixson-Crowell model, w_0 and w_t represent the drug weight at time zero and t, respectively. The k_0, k_1, k_H, k_{HC}, and k_{KP} are the release kinetic constants in zero-order, first-order, the Higuchi, Hixson–Crowell, and Korsmeyer-Peppas models, respectively. In the Weibull model, a and b variables are constants. In the Korsmeyer-Peppas, the n variable is the release exponent, indicating the drug's release mechanism. The results of the fitting are summarized in Table S2.

The results indicate that the relevant data are well fitted to the Korsmeyer-Peppas model. So, the release exponent in Korsmeyer–Peppas model is calculated. Accordingly, the value of release exponent explore the release mechanism since n = 0.5, 0.5 < n < 1, n = 1 and n > 1 representing Fickian diffusion, anomalous (non-Fickian) diffusion (i.e. by both diffusion and erosion [11]), case II transport (zero-order (time-independent) release) and super case II transport, respectively [10]. Accordingly, due to the values of release exponent in the Korsmeyer-Peppas model, which are obtained between 0.5 and 1, the anomalous (non-Fickian) diffusion is the mechanism of cisplatin release from MS1, MS2, and MS3 hydrogels. The results are summarized in Table S3. According to the cisplatin release mechanism from MS1, MS2, and MS3 hydrogels, which is accompanied by diffusion and erosion processes; the Kopcha release model is used to explore the exact contribution of diffusion and erosion using the Eq. 7 [12]:

$$M_t = A\sqrt{t} + Bt \tag{7}$$

Where t represents the release time; A and B represent the diffusion and erosion terms, respectively. The contribution of diffusion and erosion in the release mechanism is demonstrated by A to B ratio. The contribution ratio is shown in forms of A/B=1, A/B>1, and A/B<1, describing as equal contribution between diffusion and erosion, the diffusion predominates over erosion, and the erosion predominates over diffusion, respectively [9–11,13]. The obtained parameters of the Kopcha release model are summarized in Table S3. According to the results, the non-Fickian process occurred for the cisplatin release from MS1, MS2, and MS3 hydrogels. Also, the Kopcha release model parameters show no lag time and an explosion in the release mechanism since the values of the A parameter are not obtained significantly negative or positive. Moreover, the A/B parameter indicates that erosion is predominant over diffusion for cisplatin release from MS1 and MS2 hydrogels. In the case of MS3 hydrogel, diffusion is dominant over erosion.

Table S2: The mathematical kinetics models correlation coefficient for cisplatin release from MS1, MS2, and MS3 hydrogels.

**Sample code**	**Zero-order**	**First-order**	**Higuchi**	**Hixson-Crowell**	**Weibull**	**Korsmeyer-Peppas**
	**R^2**	**R^2**	**R^2**	**R^2**	**R^2**	**R^2**
**MS1**	0.85	0.89	0.92	0.89	0.89	0.98
**MS2**	0.84	0.91	0.94	0.88	0.91	0.98
**MS3**	0.88	0.86	0.95	0.88	0.88	0.97

Table S3: The release exponent parameter and Kopcha release model parameters for cisplatin release from MS1, MS2, and MS3 hydrogels.

**Sample code**	**Korsmeyer-Peppas**		**Kopcha**		
	**n**	**R^2**	**A(μg hour$^{-1/2}$)**	**B(μg hour^{-1})**	**A/B(hour$^{1/2}$)**
**MS1**	0.72	0.97	0.07	0.12	0.58
**MS2**	0.69	0.98	0.11	0.13	0.85
**MS3**	0.75	0.98	0.18	0.16	1.13

- *Protein interaction*

Herein, the interaction mechanism of the MS2 hydrogel with the model proteins is investigated using the intrinsic fluorescence changes. As shown in Fig S2, the intrinsic fluorescence intensities of HSA and HB are regularly decreased with each addition of MS2 hydrogel at 25°C and 37 °C. Hence, to indicate the mechanism of quenching, the Stern-Volmer equation (Eq. 8) is used as follows:

$$\frac{F_0}{F} = 1 + K_{SV}[Q] = 1 + k_q\tau_0[Q] \quad (8)$$

Where F_0, F, K_{SV}, k_q, τ_0, and [Q] are the fluorescence intensity in the absence of quencher, the fluorescence intensity in the presence of quencher, the Stern-Volmer quenching constant, the biomolecular quenching rate constant, the average lifetime of the fluorophore (here Trp) in the absence of quencher, and concentration of quencher, respectively.

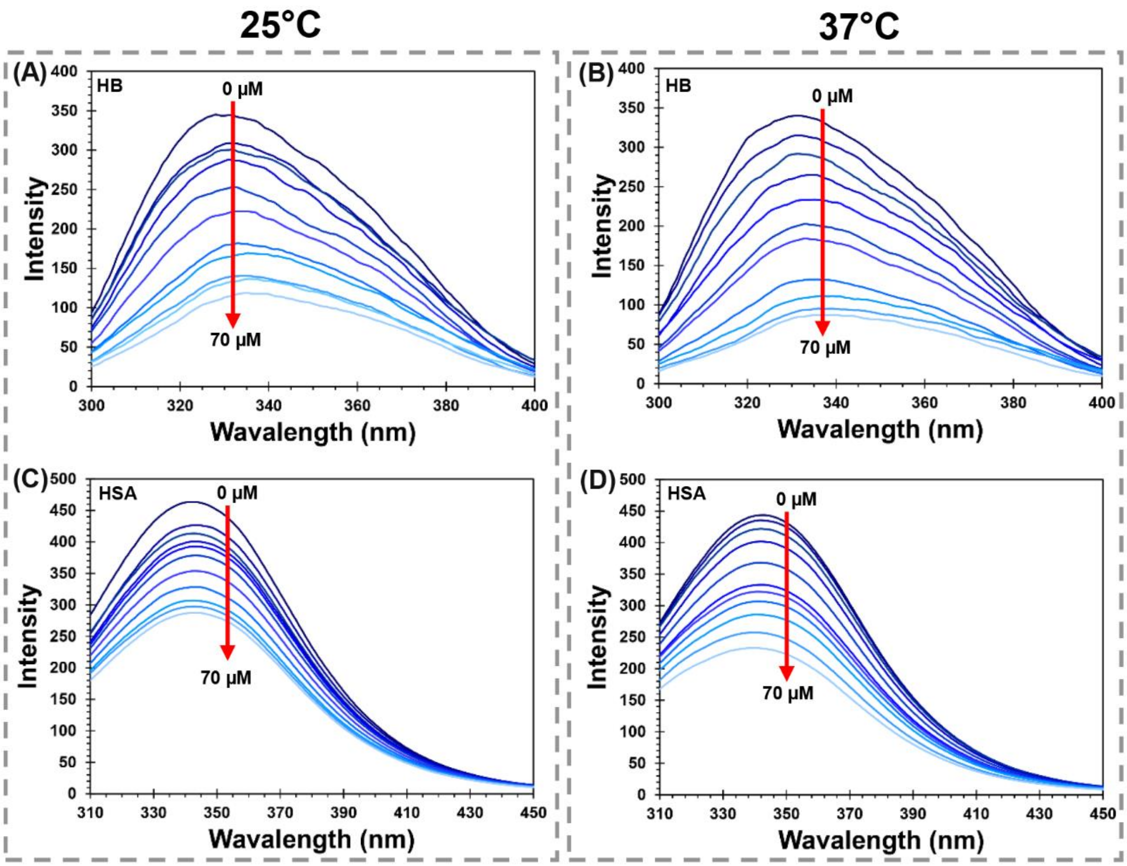

(Fig. S2) Fluorescence emission spectra changes of HB and HSA by MS2 hydrogel. A and B show the fluorescence quenching of HB (6 μM) in the presence of MS2 hydrogel (0-70 μM) at 25 and 37 °C, respectively. C and D show the fluorescence quenching of HSA (6 μM) in the presence of MS2 hydrogel (0-70 μM) at 25 and 37 °C, respectively.

The quenching mode is a combination of static and dynamic since the Stern-Volmer plot for both proteins is non-linear at 25 and 37 °C (Fig. S3 (A, B)). The graphs have upward curvature revealing that one quenching mechanism is dominated during complex formation [14]. Besides, it means rising temperature drives the quenching mechanism toward the dynamic. Nevertheless, it shows that the complex formation between the hydrogel and the model proteins is unstable with

increasing temperature. The slope deviation of graphs demonstrates that hydrogel interacts with the model proteins differently, which leads to the distinct complex stability for HSA and HB. It is caused by the chemical structure of the hydrogel and the model proteins' surface complementary properties with the hydrogel. There is an insight that the hydrogel has complementary features with the surface of the model proteins as the critical proteins of blood in terms of biocompatibility property and can make a favorable complex to boost the practical goal.

However, to address the precise contribution of the quenching parameters in the combined quenching mechanism according to the positive deviation in the Stern-Volmer plot, the Modified Stern-Volmer equation (Eq. 9) is used as follows:

$$\frac{F_0}{\Delta F} = \frac{F_0}{F_0 - F} = \frac{1}{f_a K_{SV}} \frac{1}{[Q]} + \frac{1}{f_a} \qquad (9)$$

In this regard, f_a is the fraction of accessible fluorophores [15]. The results are shown in Fig. S3 (C, D) and Table S4. The f_a is 0.97 and 0.59 for the HSA-hydrogel complex at 25 and 37 °C, respectively. It reveals that owing to HSA conformational changes, fewer Tryptophan residues are accessible for the hydrogel with increasing temperature. The hydrogel accessibility to β-Trp37 rises from 0.91 to 0.98 for the HB-hydrogel complex at 25 and 37 °C, respectively. It demonstrates that more fluorophores are exposed to the hydrogel due to HB conformational changes. It suggests that the hydrogel chemical structure changes the tryptophan residue location in the model proteins structure. Thus, the findings are coherent with the Stern-Volmer plot according to the different complex formations due to the hydrogel chemical structure and the surface properties of the model proteins.

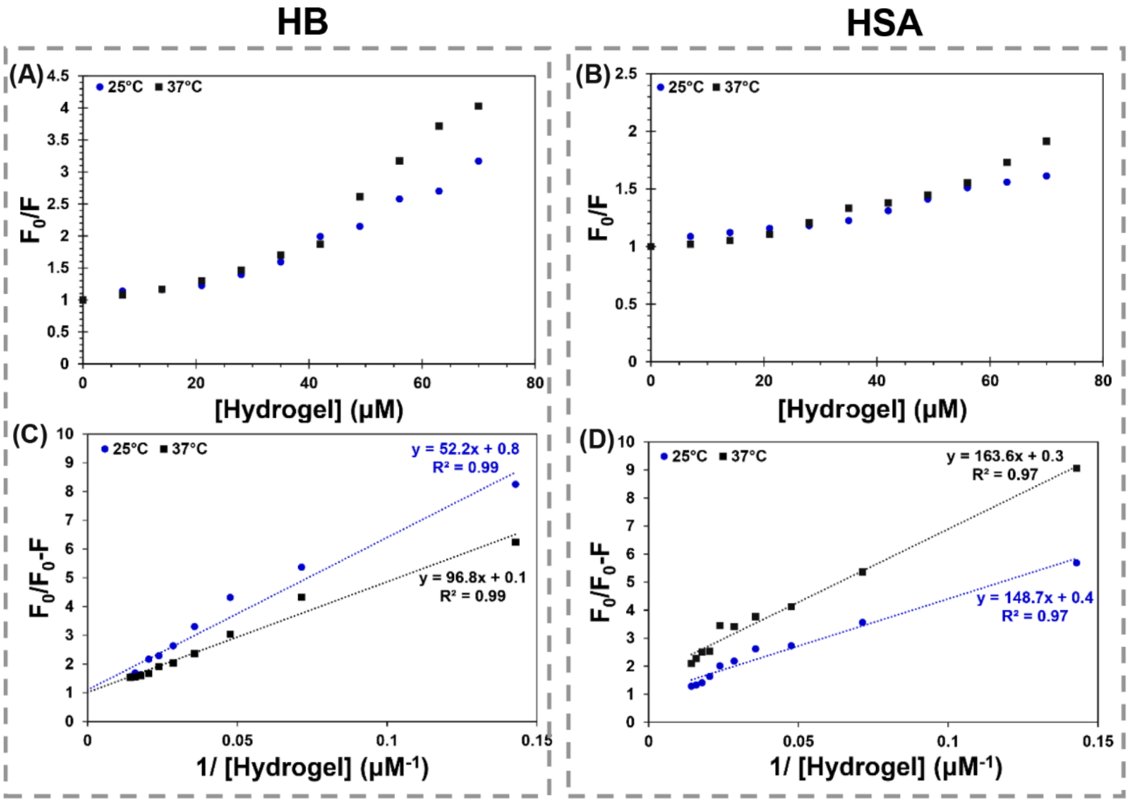

(Fig. S3) A and B indicate the Stern-Volmer plot of MS2 hydrogel interaction with HB and HSA at different temperatures, respectively. C and D show the modified Stern-Volmer plot of MS2 hydrogel interaction with HB and HSA, respectively.

The constant K_{SV} in all cases increases with rising temperature. The obtained values rate changes are due to the model proteins' conformational alterations through complex formation with the hydrogel, leading to the compensating effects of dynamic quenching and static quenching on each other. The findings fully correspond with the Stern-Volmer plots, demonstrating that dynamic quenching contribution increases slightly with increasing temperature. Nonetheless, according to the compensatory behavior of the quenching mechanism and alterations in f_a and K_{SV}, the biomolecular quenching rate constant determines the interaction mechanism. The obtained values of constant k_q are more than the maximum diffusion rate of molecules (2×10^{10} $M^{-1}s^{-1}$) in the

hydrogel interaction with the model proteins at 25 and 37 °C (Table S4). It elucidates that static quenching is dominant in the quenching mechanism. Therefore, a complex is formed between the hydrogel and the model proteins, which rising temperature causes complex instability.

Table S4: Quenching parameters for the hydrogel interaction with the model proteins.

**Protein**	**Temperature (°C)**	**f_a**	**K_{sv} ($\times 10^6$ M^{-1})**	**k_q ($\times 10^{12}$ $M^{-1}s^{-1}$)**
**HSA**	25	0.97	0.031	3.1
	37	0.59	0.032	3.2
**Hb**	25	0.91	0.021	2.1
	37	0.98	0.027	2.7

References:

[1] S.H. Jeong, Y.F. Fan, J.U. Baek, J. Song, T.H. Choi, S.W. Kim, H.E. Kim, Long-lasting and bioactive hyaluronic acid-hydroxyapatite composite hydrogels for injectable dermal fillers: Physical properties and in vivo durability, J. Biomater. Appl. 31 (2016) 464–474. doi:10.1177/0885328216648809.

[2] P.G. de Gennes, Polymers at an interface; a simplified view, Adv. Colloid Interface Sci. 27 (1987) 189–209. doi:10.1016/0001-8686(87)85003-0.

[3] P.G. De Gennes, Polymers at an interface. 2. Interaction between two plates carrying adsorbed polymer layers, Macromolecules. 15 (1982) 492–500. doi:10.1021/ma00230a055.

[4] S. Merabia, P. Sotta, D.R. Long, Unique plastic and recovery behavior of nanofilled elastomers and thermoplastic elastomers (Payne and Mullins effects), J. Polym. Sci. Part B Polym. Phys. 48 (2010) 1495–1508. doi:10.1002/polb.22046.

[5] A.S. Sarvestani, X. He, E. Jabbari, The role of filler-matrix interaction on viscoelastic response of biomimetic nanocomposite hydrogels, J. Nanomater. 2008 (2008) 1–9. doi:10.1155/2008/126803.

[6] P. Costa, J.M. Sousa Lobo, Modeling and comparison of dissolution profiles, Eur. J. Pharm. Sci. 13 (2001) 123–133. doi:10.1016/S0928-0987(01)00095-1.

[7] K. Kosmidis, P. Argyrakis, P. Macheras, A reappraisal of drug release laws using Monte Carlo simulations: the prevalence of the Weibull function, Pharm. Res. 20 (2003) 988–995.

[8] T. Higuchi, Mechanism of sustained-action medication. Theoretical analysis of rate of release of solid drugs dispersed in solid matrices, J. Pharm. Sci. 52 (1963) 1145–1149.

[9] A.W. Hixson, J.H. Crowell, Dependence of Reaction Velocity upon surface and Agitation, Ind. Eng. Chem. 23 (1931) 923–931. doi:10.1021/ie50260a018.

[10] R.W. Korsmeyer, R. Gurny, E. Doelker, P. Buri, N.A. Peppas, Mechanisms of solute release from porous hydrophilic polymers, Int. J. Pharm. 15 (1983) 25–35. doi:10.1016/0378-5173(83)90064-9.

[11] S. Prodduturi, K.L. Urman, J.U. Otaigbe, M.A. Repka, Stabilization of hot-melt extrusion formulations containing solid solutions using polymer blends, AAPS PharmSciTech. 8 (2007) E152–E161. doi:10.1208/pt0802050.

[12] M. Kopcha, K.J. Tojo, N.G. Lordi, Evaluation of Methodology for Assessing Release Characteristics of Thermosoftening Vehicles, J. Pharm. Pharmacol. 42 (1990) 745–751. doi:10.1111/j.2042-7158.1990.tb07014.x.

[13] S. Thumma, S. Majumdar, M.A. ElSohly, W. Gul, M.A. Repka, Preformulation Studies of a Prodrug of Δ9-Tetrahydrocannabinol, AAPS PharmSciTech. 9 (2008) 982–990. doi:10.1208/s12249-008-9136-7.

[14] B. Ghalandari, K. Asadollahi, A. Shakerizadeh, A. Komeili, G. Riazi, S.K. Kamrava, N. Attaran, Microtubule network as a potential candidate for targeting by gold nanoparticle-assisted photothermal therapy, J. Photochem. Photobiol. B Biol. 192 (2019) 131–140. doi:10.1016/j.jphotobiol.2019.01.012.

[15] S. Nourizadeh, A. Divsalar, M. Fekri, B. Ghalandari, M. Eslami-Moghadam, A. Akbar Saboury, S. Abbasi, Multiple Spectroscopic, Docking and Cytotoxic Study of a Synthesized 2,2′ Bipyridin Phenyl Isopentylglycin Pt(II) Nitrate Complex: Human Serum Albumin and Breast Cancer Cell Line of MDA-MB231 as Targets, J. Fluoresc. 28 (2018) 551–559.

doi:10.1007/s10895-018-2216-5.
